# Grafting of Human Bone

**Published:** 1887-08

**Authors:** 


### THE GRAFTING OF HUMAN BONE.

ON the 25th of November, 1886, a young man, seventeen years of age, had a complicated fracture of the right leg, about its middle, from which escaped a considerable fragment of the superior part. Succeeding this there were severe phlegmonous complications, the fractured extremities necrosed and were eliminated, resulting in a considerable loss of bone. In December, 1886, the fracture had not united, and the bones were from 35 to 40 milimeters (about 1½ inches) apart, with a fibrous cord uniting them. The fibula, which was intact, formed a splint for the tibia, and had prevented a union of the osseous extremities. M. Poncet then tried the experiment of an osseous graft, and executed it in the following manner: From a leg which had just been amputated, on account of traumatism, he took the first phalanx of the great toe, dissected away its cartilaginous surfaces, getting a fragment one inch in length, which he antisepticized in a tepid solution of corrosive sublimate of one to two thousand. The pseudarthrosis having been exposed, M. Poncet cut away the fibrous bridge, polished the two bones, and placed the osseous graft in the fibrous tissue so as to bring it in contact with the inferior fragment of the tibia. There was no reunion; careful antiseptic dressing was continued. Sixty-two days after the operation the graft was completely covered with fleshy granulations, except in a space of three to four square centimeters in the middle, where the bone appeared of a pale pink hue. The mobility of the pseudarthrosis persisting, M. Poncet decided to make a resection of the osseous suture. In performing this operation he removed the graft from the attached portions of the bone, and observed the following important facts: The graft was intimately united to the tibia at one of its extremities by a somewhat dense, tough, fibrous nonosseous tissue. Its entire periphery was covered by the fibrous tissue and a layer of more and more dense granulations. The bone itself was perfectly vascular and alive, eroded at some points by the granulations heaped against the surface. This result shows that legitimate hopes may be built upon this method.—*Le Concours Medical.*

